# [18 F]-Fluoroestradiol PET (FES-PET) and [18 F] Flurodeoxyglucose PET (FDG-PET) Imaging May Aid in Managing Therapy in Patients with Metastatic Lobular Breast Cancer

**DOI:** 10.1007/s11307-025-02015-2

**Published:** 2025-05-14

**Authors:** Poorni M. Manohar, Lanell M. Peterson, Isaac C. Jenkins, Qian Vicky Wu, Brenda F. Kurland, Alena Novakova-Jiresova, Mark Muzi, Delphine L. Chen, Jennifer M. Specht, Suzanne Dintzis, Paul E. Kinahan, David A. Mankoff, Hannah M. Linden

**Affiliations:** 1 University of Washington, Seattle, WA USA; 2 Fred Hutchinson Cancer Research Center, FHCC 1144 Eastlake (LG-200), Seattle, WA 98109-1023 USA; 3 GSK, Philadelphia, PA USA; 4 Charles University and Thomayer Hospital, Prague, Czech Republic; 5 University of Pennsylvania, Philadelphia, PA USA

**Keywords:** FES, FDG, Lobular breast cancer

## Abstract

**Aim:**

This study examines the combination of FES-PET and FDG-PET as complementary imaging for detection of metastatic ILC.

**Methods:**

We retrospectively evaluated FES and FDG uptake in patients with metastatic ILC from an estrogen receptor (ER) positive primary tumor. We classified lesions into three categories (FES high/FDG low, FES high/FDG high, FES low/FDG low) using SUVmax cut-off values of 1.5 for FES and 5.0 for FDG. Qualitative evaluation included examination of the difference in the extent of disease between FES and FDG.

**Results:**

Of the 38 patients, 82% had FES uptake in all tumor sites identified by FDG, with 18% lacking FES uptake in at least one lesion. Mean (range) SUVmax for FES and FDG was 4.0 (0.67–10.6) and 4.6 (1.3–12.5), respectively. The majority of ILC patients (25/38), had lesions with FES high/FDG low uptake, consistent with the strongly ER + indolent nature of ILC. Patients with disease classified as FES high/FDG low had longer median overall survival (OS) (3.2 years) and progression-free survival (PFS) (1.5 years) than FES high/FDG high (OS = 2.1 years and PFS = 0.46 years). The median overall OS for all patients was 3.0 years (95% CI 2.5, 4.8) and PFS of 1.3 years (95% CI 0.6, 2.5). 8 patients (21%) had qualitatively more extensive disease by FES-PET.

**Conclusions:**

Our findings suggest that both FES-PET and FDG-PET can identify metastatic ILC and be useful in detecting the pattern and extent of disease. The imaging combination provides additional information for prognosis and clinical decision making, balancing suitability for endocrine therapy and aggressiveness/indolence of disease.

**Supplementary Information:**

The online version contains supplementary material available at 10.1007/s11307-025-02015-2.

## Introduction

Approximately 155,000 women are living with metastatic breast cancer in the United States [1]. Infiltrating Lobular Cancer (ILC), comprising 5–15% of breast cancers, is a different biological entity from the more common ductal histology [2, 3]. Of the few studies focused on lobular breast cancer, the majority target early breast cancer therapies, leaving a knowledge gap in metastatic lobular carcinoma [4, 5]. Tumors of lobular histology tend to be indolent and diffuse with a predilection for serosal surfaces as metastatic sites such as the gastrointestinal tract, ovaries, peritoneum, retroperitoneum, peri-orbital and leptomeninges [6]. These distinct sites and net-like pattern of spread can make extent of disease difficult to assess by conventional imaging, including by FDG-PET [7–9].

Establishing hormone receptor status at the time of diagnosis and recurrence is vital in selecting appropriate breast cancer therapy [10]. Discrepant estrogen receptor expression (ER) between the primary and metastatic site in up to 20% of patients emphasizes the need to confirm ER status with metastatic biopsy [11, 12]. However, current practice of metastatic biopsy has many limitations, including challenging biopsy sites, identification of healing or normal bone, and sampling that precludes assessment of intra-tumoral heterogeneity [13, 14]. ILC is over-represented in western countries, and in late reccurence and de novo metastatic disease. Patients with ER positive breast cancer, regardless of histology, will develop resistance and progression following initial endocrine therapy highlighting the value of an imaging assay of hormone receptor expression [15, 16]. The treatment of ER + metastatic breast cancer is improving; we have better outcomes with molecularly targeted agents in synergy with endocrine therapy, and these benefits extend to ILC.

There is an increasing clinical use of PET imaging in breast cancer [17]. 2-deoxy-2-[^18^F]fluoro-D-glucose (FDG), a glucose analog, has long been the most widely used clinical PET tracer to detect metabolic activity in tumors. 16-alpha-^18^F-fluoro-17-β-estradiol (FES) is an estrogen analogue that can detect ER binding throughout the body and provide simultaneous assessment of multiple lesions and was approved for clinical use in the United States in 2020 (NDA 212155). FES uptake strongly correlates with ER expression with high sensitivity (83%) and specificity (83%) for detection of ER-positive breast cancer [18] FES-PET appears stable over a course of therapy within each patient [19] and has shown promise in detecting heterogeneity in breast cancer, which has clinical prognostic and treatment implications in addition to improved insight into tumor biology [20, 21]. It has also been evaluated in small studies as a feasible and effective predictive biomarker to predict clinical benefit of endocrine therapy [18, 22–26]. In particular, case reports document the value of FES-PET in metastatic ILC and demonstrate its ability to impact clinical care, especially in settings where conventional imaging is inconclusive [27]. A retrospective review of 7 patients with FES-PET and FDG-PET scans provides initial insights into the differences between two scan modalities. Head-to-head comparison revealed more lesions visible on FES-PET than FDG-PET and one patient with a metastatic lesion detected on FES that was not identified by FDG [28]. Recently, in a retrospective analysis of 20 patients, FES-PET was shown to be more effective than FDG-PET in detecting bone metastasis in ILC [29]. A prospective pilot study to assess the frequency with which sites of histologically proven ILC have abnormal uptake on FES-PET found the frequency to be significantly greater than 60% [30].

Our study expands on the growing literature of FES-PET in metastatic ILC. The purpose of this study was to examine paired FES-PET and FDG-PET imaging to show that together they are complementary and useful in detection of metastatic ILC. We examined a historical cohort in our multi-study database to characterize ILC lesions, and study outcomes.

## Methods

### Patient Selection

**Fig. 1:**
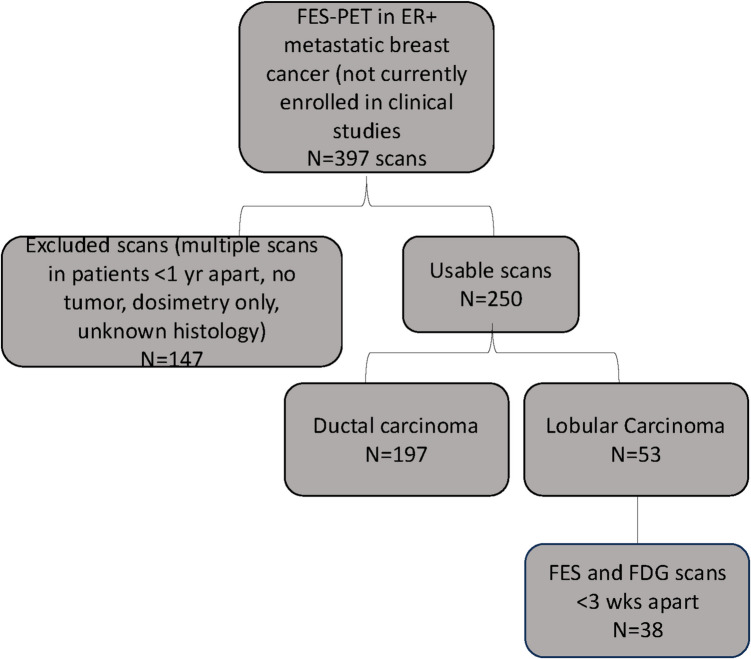
STARD diagram for inclusion in FES lobular disease study

This was a retrospective review of patients with ER positive metastatic breast cancer enrolled in various prospective studies from 1996 to 2015. Patients with lobular disease scanned after this time are part of ongoing clinical trials and the data cannot be used until the trials have been completed. FES-PET was performed for 397 metastatic breast cancer patients at the time of diagnosis or during later lines of therapy. All patients had ER positive disease by immunohistochemistry, based on evaluation of available tissue, either primary site or distant metastases. Patients with ductal histology, unusable data, scans used for dosimetry, or unknown histology were excluded. Metastatic biopsies with reports of both ductal and lobular features were re-assessed by a pathologist specializing in breast cancer to categorize a definitive histology. All patient data has been used in previously published studies. A total of 38 patients were identified with lobular histology. Figure 1 shows the Standards for Reporting Diagnostic accuracy studies (STARD) diagram for patient selection.

### PET Imaging

Patients underwent FDG-PET and FES-PET studies before or shortly following the initiation of standard-of-care endocrine therapy with no more than 3 weeks and no change in therapy between scans. FDG-PET was performed for clinical indications or as part of a research study. All patients were off endocrine blocking therapy at the time of FES-PET imaging and/or had a miniumum of 4 weeks for endocrine blocking therapy washout. Whole-body imaging was performed by scanning multiple fields-of-view (FOV) on either a PET scanner (GE Advance) or PET/CT (GE Discovery STE) scanner operating in high sensitivity mode. The scanners were cross-calibrated and monitored with a NIST-traceable reference source [31, 32].

^18^F-FDG was prepared in-house or purchased commercially from Cardinal Health (Seattle, WA). All FDG-PET imaging was performed according to institutional clinical protocol, including > 6 h fasting.

^18^F-FES was prepared according to published methods (IND#79,005) [33]. The injected doses were within imaging recommendations of 111 MBq to 222 MBq (3 mCi to 6 mCi). FES-PET imaging consisted of a single FOV 60-min dynamic scan (data not used for this study) immediately followed by a 4–5 FOV torso scan [34]. If lesions were suspected by additional imaging, more FOVs could be added. The FES-PET torso scan used the same imaging parameters as the FDG-PET scan. Fasting was not required.

### Image Analysis

For FDG-PET studies, Standardized Uptake Values (SUVmax) from previously generated regions-of-interest (ROIs), over the area of the lesions reflecting the most active areas of disease, were used on up to 10 tumor sites per study, as described [35]. If there was any concern that uptake in areas on the FDG scans were not metastatic disease, the lesions were validated by additional imaging (i.e. CT or bone scan). Quantitative analysis of FES-PET images used 173 lesions matched from the FDG-PET images drawn on the torso scan from earlier studies, as previously defined [36]. No new lesion ROIs were drawn. Lesional FES-PET was considered positive if the lesion detected on the index FDG scan was above the blood pool background by the FES-PET scan, but may not be categorized as FES high. In order for the overall scan to be read as positive, all lesions had to be considered positive. Each patient had only one set of scans.

Qualitative analysis visually compared the FES-PET and FDG-PET images side-by-side. If the FES-PET scan appeared to have a more extensive pattern of spread (i.e. more lesions) and hence lesions easier to identify than the FDG-PET, it was considered positive.

### Statistical Analysis

To define classification categories, we chose a cut-off value for FES (SUVmax of 1.5) and a conservative cut-off value for FDG (SUVmax of 5) based on previous studies and recent publications that included both ductal and lobular carcinomas [28, 30, 35–38] noting the differences in using lean body mass corrected SUV (SUL) and in reducing the number of categories. Clinically, only SUVmax is used in imaging reports, and we used 3 categories instead of 4. Lesions were classified as FES high/FDG low, FES high/FDG high, and FES low/FDG low. Only one lesion was classified as FES low/FDG high (FES = 1.47, FDG = 5.02), but since the FDG value was a borderline case, it was reclassified as FES low/FDG low. Patients were assigned a representative classification group by averaging all FES and FDG values within a patient and then applying the cutoffs to those averages. Primary analyses focused on patient level comparisons of these classification groups (n = 38 patients). A secondary, exploratory analysis compared bone marrow uptake among a subset of patients with bone dominant (no visceral organ involvement) metastatic disease (n = 18). Only 3 of those patients had diffuse bone marrow uptake. Differences in demographics, clinical, uptake and imaging parameters were assessed using a Kruskal–Wallis test for continuous variables and Fisher’s exact test for categorical variables [37, 38]. Overall survival (OS), defined as time from FES-PET to death, and progression-free survival (PFS), defined as time from FES-PET to disease progression or death whichever occurs first, were evaluated using Kaplan–Meier curves and log rank tests. Hazard ratios were estimated with unadjusted Cox proportional hazards regression. For the bone dominant disease analysis, log-transformed SUVmax of all lesions (n = 110) were compared between those with and without bone marrow uptake using a simple linear mixed effects model containing a random effect to account for within patient correlation. P-values less than 0.05 were considered statistically significant and all tests were two-sided. Analyses were performed using the statistical programming language R (version 4.2).

## Results

Demographics and clinical characteristics for the full ILC cohort and subsets of patients with bone metastasis and bone marrow disease are summarized in Table 1. The median age of the patients was 59 years at the time of the first FES scan. All identified patients were female with pathology proven metastatic invasive lobular breast cancer. The most common site of metastases was bone, followed by lymph nodes. Eighteen patients were found to have bone dominant disease, including 3 with diffuse marrow involvement. FES-PET identified lesions in bone, soft tissues, bone marrow, and lung. Patients received a variety of therapies including endocrine, chemotherapy, and radiation prior to the first imaging scan and the majority of patients received endocrine therapy following the FES scan (31/38–82%). Differences in demographics, clinical, uptake and imaging characteristics were not significant (data not shown).

**Table 1:** Baseline demographics

	All (N = 38)	Bone Mets (N = 15)	Bone Marrow (N = 3)
Age at FES, years			
Mean (SD)	60 (11)	59 (11)	46 (10)
Range	35–80	39–74	35–54
Sex			
Female (%)	38 (100)	15 (100)	3 (100)
Primary lesion ER^*^			
Positive (%)	38 (100)	15 (100)	3 (100)
Primary lesion HER2			
Positive (%)	3 (8)	1 (7)	1 (33)
Negative (%)	31 (82)	14 (93)	2 (67)
Unknown or Indeterminate (%)	4 (10)	0 (0)	0 (0)
FES^†^			
Positive (%)	31 (82)	14 (93)	3 (100)
Negative (%)	5 (13)	1 (7)	0 (0)
Heterogeneous (%)	2 (5)	0 (0)	0 (0)
Prior Hormone Therapy (%)	26 (68)	13 (87)	1 (33)
Prior Chemotherapy (%)	26 (68)	12 (80)	2 (67)
Prior Radiation Therapy (%)	20 (53)	10 (67)	1 (33)
Endocrine Refractory^‡^			
Yes (%)	5 (13)	2 (13)	1 (33)
No (%)	20 (53)	11 (73)	1 (33)
Unknown (%)	13 (34)	2 (13)	1 (33)
Molecular Subtype			
Luminal A (ER +/PR +)	26 (68)	11 (73)	2 (67)
Luminal A (ER +/PR-)	8 (21)	2 (13)	1 (33)
Unknown (PR Missing)	4 (11)	2 (13)	0 (0)
Primary Tumor Grade			
1	10 (26)	3 (20)	1 (33)
2 +	10 (26)	3 (20)	2 (67)
Unknown	18 (48)	9 (60)	0 (0)
Ki67%			
0–25%	12 (32)	3 (20)	2 (67)
> 25%	5 (13)	3 (20)	1 (33)
Unknown	21 (55)	9 (60)	0 (0)
Metastatic Site Location^§^			
Bone (%)	26 (68)	15 (100)	3 (100)
Lymph Nodes/Soft Tissue (%)	15 (39)	0 (0)	0 (0)
Breast (%)	6 (16)	0 (0)	0 (0)
Lung (%)	1 (3)	0 (0)	0 (0)
Post FES Therapy			
Endocrine (%)	31 (82)	14 (93)	1 (33)
Chemotherapy (%)	3 (8)	1 (7)	2 (67)
Other or unknown (%)	4 (10)	0 (0)	0 (0)

^*^IHC were reviewed from most recent available data to determine hormone status

^†^FES-PET was considered positive if all lesions detected on the FDG-PET scan were evaluable above background levels on the FES-PET scan, negative if all lesions detected on the FDG-PET scan were not above background on the FES-PET scan and heterogeneous if at least one lesion on the FES-PET scan was below background in an otherwise positive scan

^‡^Endocrine Refractory is defined as transition to chemotherapy after at least 1 line of endocrine therapy for metastatic breast cancer treatment

^§^ For the site variables (e.g., Bone), the N(%) indicates the number of patients that had at least one lesion identified at that site by FES-PET and/or FDG-PET

Table 2 shows the dose and PET image timing for both the FES and FDG scans. Most of the scans were done on the same scanner with the exception of 4 studies where the clinical FDG scan was done on the DSTE scanner and the FES was done on the Advance.

**Table 2:** Summary of FES and FDG dose and imaging parameters

	Average Dose (MBq) (range)	Average Uptake time (min) (range)	Advance PET	DSTE PET
FES	188.7 (148–244.2)	71.5 (61–107)	33	5
FDG	366.3 (284.9–403.3)	50.5 (35–71.7)	29	9

### FES Qualitative Results

**Fig. 2:**
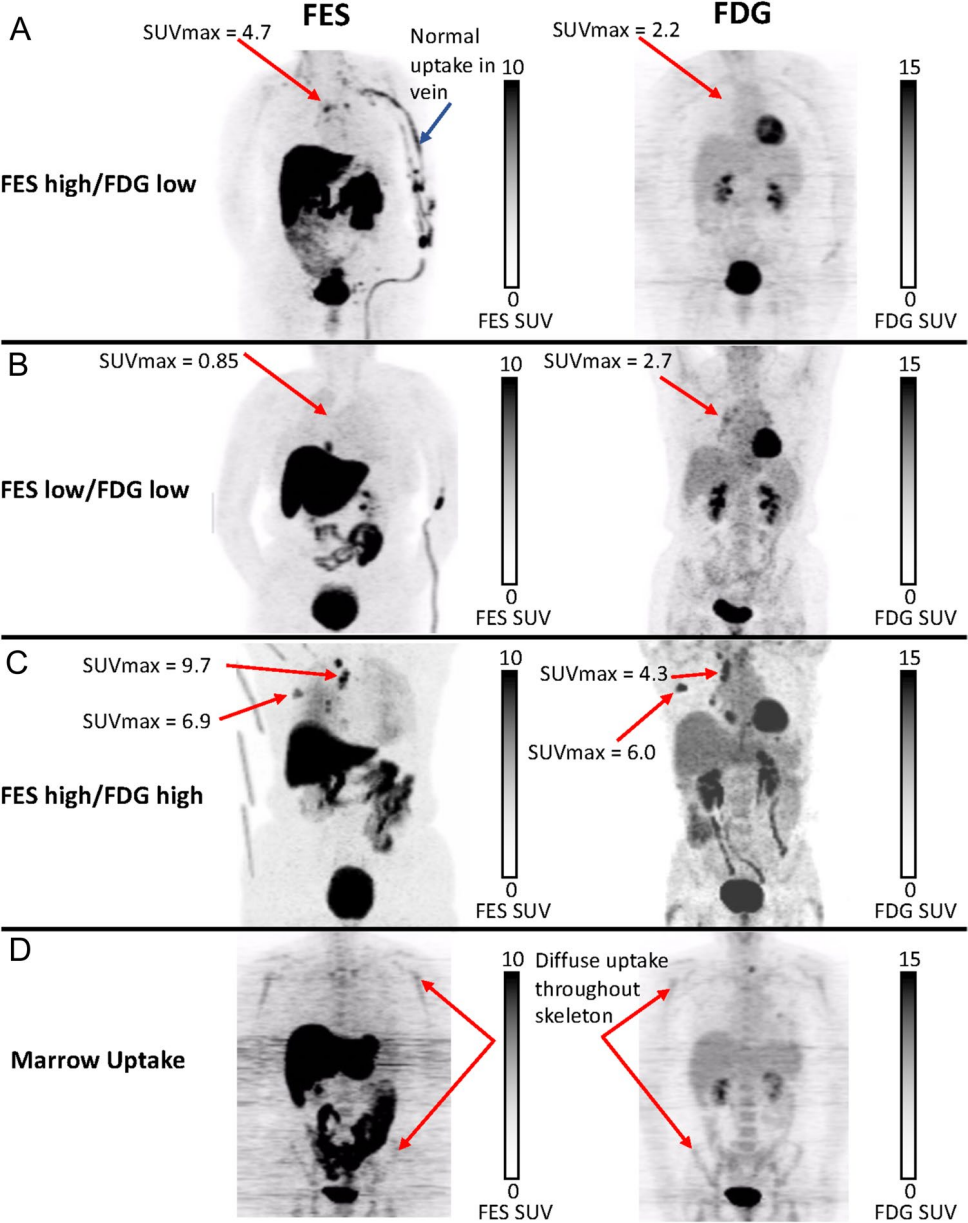
Representative 5 FOV composite (MIP) torso images from the 3 cutoff groups and a patient with diffuse bone marrow uptake. FES-PET images were obtained immediately following a single 60-min FOV dynamic scan with 5 min of static imaging per axial FOV. FDG-PET images were obtained starting approximately 45 min after injection with 5 min of static imaging per axial FOV. Panel **A** shows uptake with FES, but not FDG. Panel **B** shows faint uptake with FDG, but not FES. Panel **C** shows intense uptake with both FES and FDG. Each woman has nodal metastatic disease commonly seen in lobular breast cancer. Panel **D** illustrates diffuse marrow uptake throughout the skeleton in both FES and FDG

**Fig. 3.**
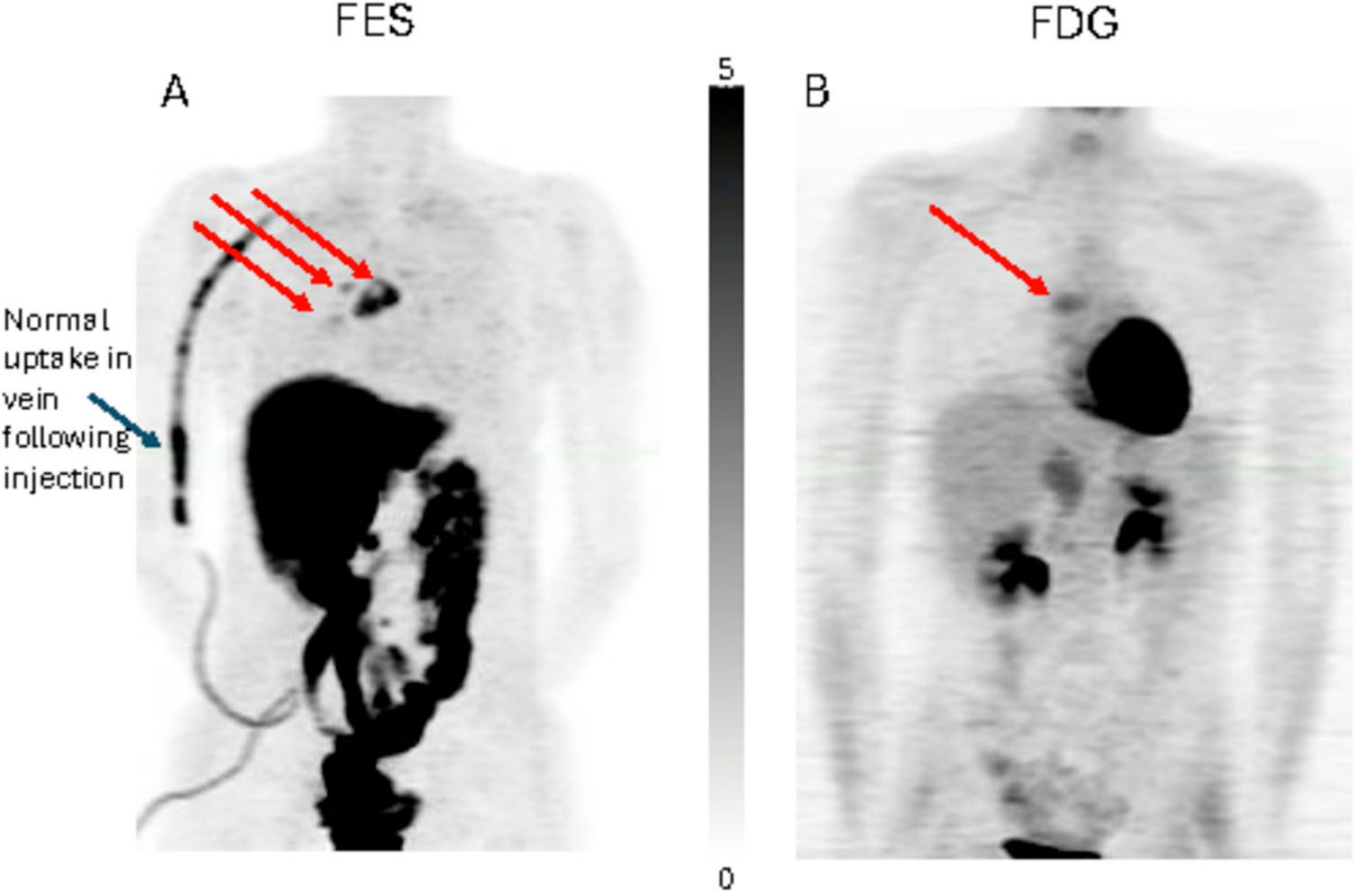
5 FOV composite (MIP) torso FES-PET and FDG-PET images. FES-PET images (**A**) were obtained immediately following a single 60-min FOV dynamic scan with 5 min of static imaging per axial FOV. FDG-PET images (**B**) were obtained starting approximately 45 min after injection with 5 min of static imaging per axial FOV. The patient is a 39-year old, premenopausal female with more extensive nodal disease easily seen by FES-PET (red arrows at multiple tumor sites). The patient progressed on letrozole after FES-PET and died of her disease approximately 8 months after PET imaging

Figure 2 displays image examples of typical nodal spread of disease with differing FES and FDG uptake and diffuse bone marrow uptake. Overall, 31/38 (82%) ILC patients had positive scans in which all analyzed lesions had both FES and FDG uptake above background. Five (13%) had scans in which all analyzed lesion were FES-negative, and 2 (5%) had heterogeneous uptake with at least one negative lesion in an otherwise positive FES scan. Qualitatively, the extent of disease appreciated by FES-PET was noted in 8/38 (21%). In these patients with ILC, FES and FDG PET together demonstrated measurable tumor burden. Figure 3 illustrates an example of a patient that had qualitatively more extensive disease visually seen by FES-PET when comparing the side-by-side images of FES-PET and FDG-PET.

### FES Quantitative Results

**Fig. 4:**
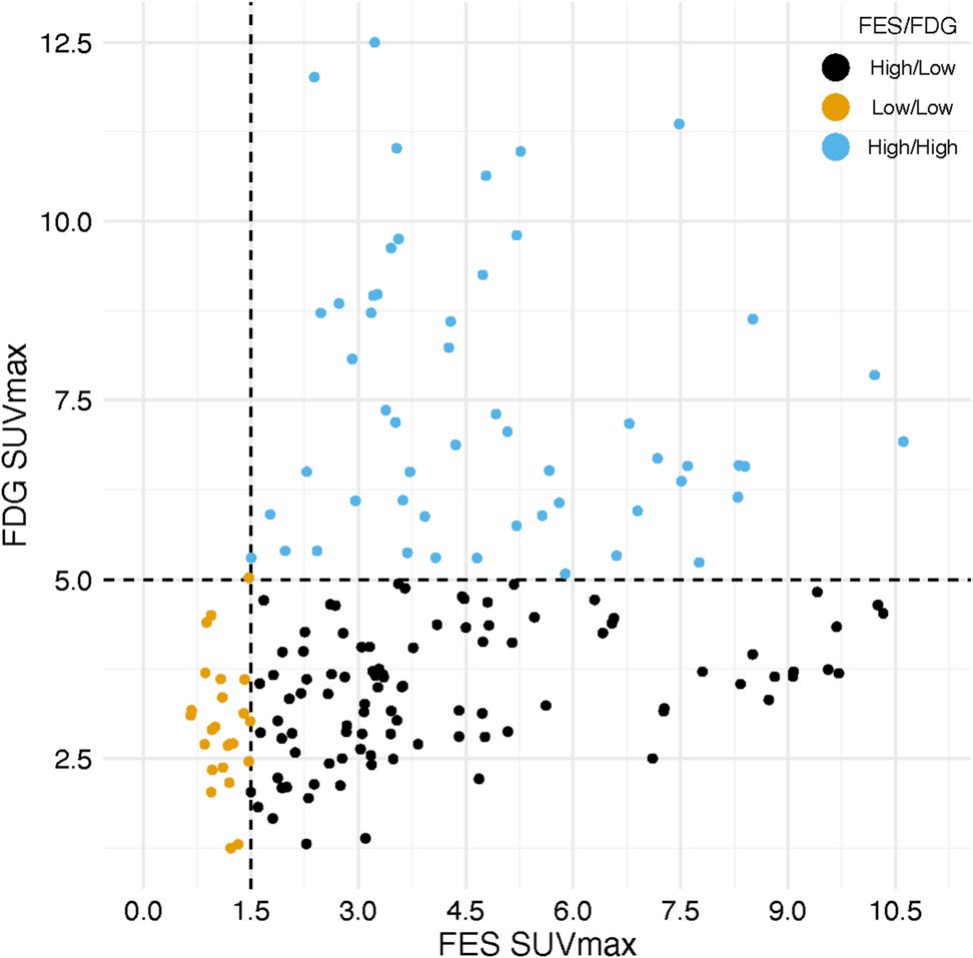
Distribution of FES-PET and FDG-PET Classification Groups. Observations and groups on the raw FES/FDG scale. The dashed lines show the cutoff values.Each patient is assigned into an FES/FDG category by averaging all FES and FDG values within a patient and then applying the cutoffs to those averages. Each patient has one set of scans (FDG-PET and FES-PET)

Among all lesions scanned, the mean (range) SUVmax for FES and FDG was 4.0 (0.66, 10.6) and 4.6 (1.3, 12.5), respectively. We found that the dominant cluster of ILC lesions fell into the FES high/FDG low sector (96/173). Mean (range) SUVmax values for FES and FDG for the three classification groups (FES high/FDG high, FES high/FDG low, and FES low/FDG low) are shown in Table 3. Lesional distribution is shown in Fig. 4. After averaging SUVmax values within patients, the majority were classified as FES High/FDG Low (20/38). Fewer than a third were classified as FES high/FDG high (11/38) and even fewer as FES low/FDG low (7/38). Although not part of this analysis, Supplemental Fig. 1 shows FES and FDG SUVmax values per lesion within each patient showing modest within patient heterogeneity (consistent with prior publications [39]).

**Table 3:** Summary of all FES and FDG scan values for the lobular cohort across FES/FDG cutoff groups

	All (N = 38)	FES high/FDG low (N = 20)	FES low/FDG low (N = 7)	FES high/FDG high (N = 11)
Lesions per patient Median (range)	3.5 (1–10)	3 (1–10)	2 (1–6)	6 (1–10)
Number of Lesions	173	85	19	69
FES SUVmax				
Mean (SD)	4.0 (2.5)	4.3 (2.5)	1.1 (0.28)	4.5 (2.2)
Median (Q1,Q3)	3.4 (2.2, 5.2)	3.5 (2.6, 5.5)	1.1 (0.95, 1.4)	3.9 (2.9, 5.6)
Range	0.66–10.6	0.96–10.3	0.66–1.6	0.86–10.6
FDG SUVmax				
Mean (SD)	4.6 (2.3)	3.5 (1.0)	2.8 (1.0)	6.5 (2.5)
Median (Q1,Q3)	4.0 (3.0, 5.8)	3.5 (2.8, 4.3)	2.7 (2.2, 3.2)	6.1 (4.7, 8.2)
Range	1.3–12.5	1.3–6.5	1.3–5.0	2.1–12.5

While not statistically different in this small group of patients, FES and FDG uptake in patients with diffuse bone marrow uptake trended higher than those with bone dominant metastatic breast cancer (Supplemental Table 1).

### Progression Free Survival (PFS) and Overall Survival (OS)

**Fig. 5:**
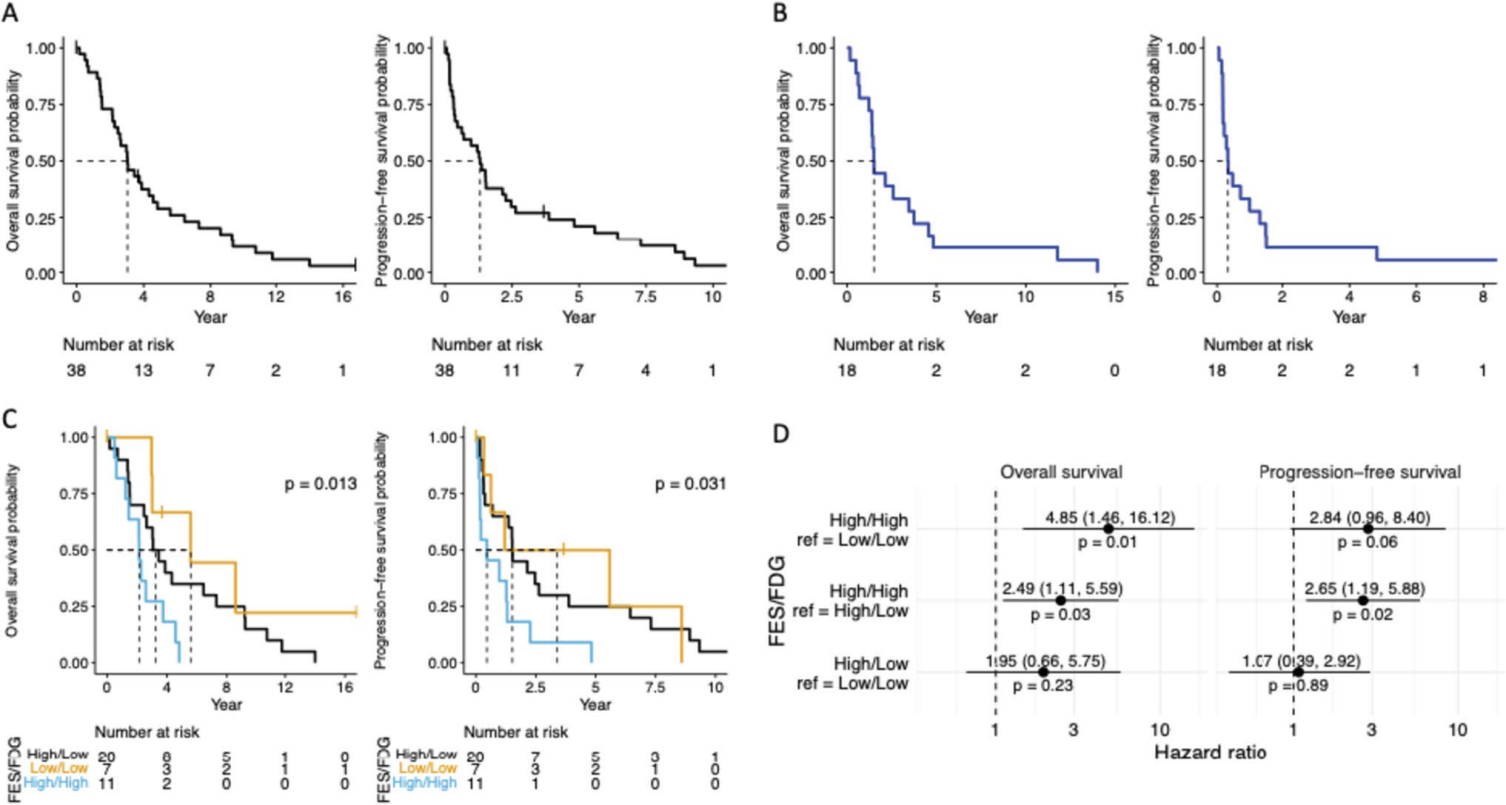
Overall Survival and Progression Free Survival. **A** Kaplan-Meier curves for overall and progression-free survival for all patients with lobular breast cancer (N = 38) **B** Kaplan-Meier curves for overall and progression-free survival among patients with bone dominant metastatic breast cancer (N = 18) **C** Kaplan-Meier curves for overall and progression-free survival among FES/FDG cutoff groups. Overall survival times are defined as time from FES scan to death or loss to follow-up. Progression-free survival times are from FES scan to death, progression, or loss to follow-up. P-value results from a Log Rank test and tests if the survival curves are different across groups (N = 20 in FES high/FDG low group, N = 7 in FES low/FDG low group, and N = 11 in FES high/FDG high group) **D** Forest plots showing the hazard ratios between FES/FDG cutoff group pairs. The reference group is on the right of the “vs” statement. Cox proportional hazard models for overall and progression-free survival used all 173 observations to assess the risk of death and/or progression across groups. Since we have repeated measurements on the patients, we include a term in the model that accounts for the within patient correlation structure

Figure 5A demonstrates the PFS and OS for patients with ILC following FES-PET imaging. Figure 5B shows the PFS and OS amongst patients with bone dominant metastatic ILC. Patients with disease classified as FES high/FDG low had longer median OS (p = 0.013) and PFS (p = 0.031) than FES high/FDG high as shown in Fig. 5C. FES high/FDG high demonstrated a higher risk of death than FES low/FDG low (HR = 4.85 [95% CI 1.46–16.12], p = 0.01) and FES high/FDG low (HR = 2.49 [95% CI 1.11–5.59], p = 0.03), as shown in Fig. 5D. Table 4 displays the PFS and OS mean and 95% Cl for each of the groups shown in Fig. 5A-5 C.

**Table 4:** Summary of OS and PFS

	All (N = 38)	Bone dominant (N = 15)	FES high/FDG low (N = 20)	FES low/FDG low (N = 7)	FES high/FDG high (N = 11)
OS (years, 95% Cl)	3.0 (2.5, 4.8)	1.5 (1.4, 4.6)	3.2 (2.5, 9.3)	5.6 (3.0, Inf)	2.1 (1.4, Inf)
PFS (years, 95% Cl)	1.3 (0.62, 2.5)	0.3 (0.17, 1.5)	1.5 (0.70, 6.4)	3.4 (0.62, Inf)	0.46 (0.17, Inf)

## Discussion

The diffuse spread of disease in lobular histology has made diagnosis, staging, and treatment selection challenging. FES-PET measures lesional concentration of estradiol at tumor sites in vivo, provides simultaneous assessment of multiple tumor sites, and predicts clinical benefit of endocrine therapies. The lesional uptake relies on spatial density of tumor cells and amount of ER expression per cell. We analyzed imaging characteristics from FES-PET and FDG-PET scans in a retrospective cohort and found that both FDG and FES PET identified lesions in metastatic lobular breast cancer.

Prior research has shown that a diagnosis of invasive lobular carcinoma carries important prognostic implications [40], however there is little knowledge about this subtype in the metastatic setting [41]. Current biomarker measurements rely on invasive methods such as biopsy and are limited by tumor heterogeneity and sampling challenges, and the distinction between ILC and IDC in metastatic lesions are rarely noted, rather the metastatic biopsy is traditionally characterized as malignancy consistent with breast cancer. FES-PET imaging may provide an option when biopsy is not feasible through functional assessment of estrogen receptor status at multiple tumor sites simultaneously. Although prior investigators have demonstrated less ability of FDG-PET to assess lobular disease [9], our study shows that both FES and FDG uptake were seen in multiple lesions in ER + metastatic lobular breast cancer. However, we note that the detection by FES and FDG-PET does not fully reflect the tumor burden, rather it detects the bulky disease which is more clinically apparent by imaging. It is likely that there is more tumor in vivo than is appreciated by qualitative and quantitative FDG-PET and FES-PET. For example, the challenge of nonspecific peritoneal and GI uptake masks the ability to detect underlying thin and sparse lesions. We found that metastatic ILC is largely characterized by lesions that are FDG-indolent and FES-avid. This suggests that FES may help identify lesions more readily, and be useful to detect functional ER expression in advanced ILC, and hence assist in therapeutic decision making over the course of long term management of disease, consistent with other studies of the application of FES-PET to invasive lobular cancer [27, 28].

Historically, the extent of disease in lobular breast cancer has been difficult to assess with FDG-PET. In our analysis, we demonstrate that across a variety of sites including bone, lymph nodes and lung, tumors of lobular histology can be evaluated by both FES-PET and FDG-PET. Our study suggests that FES-PET could be used to identify extent of disease and impact functional knowledge of ER expression. Our study also showed that many metastatic lobular cancers have modest FDG uptake, and that FDG-PET may also contribute to staging. This experience differs somewhat than other published series [27, 28] and may reflect the population included in our retrospective analysis taken from studies where most patients had previously undergone endocrine treatment of their disease and had cancer manifesting some degree of endocrine resistance.

Finally, our classification of FES and FDG uptake into categories (FES high/FDG high, FES high/FDG low, and FES low/FDG low) provides insight into how these imaging modalities are complementary and provide different information about tumor characteristics. In this cross-sectional analysis we note that that patients with FES high/FDG high scans generally have worse outcomes. This is in line with prior studies that recognize high FDG uptake as a poor prognostic feature [42–44]. Patients with FES high/FDG low scans have longer OS and PFS on average following endocrine therapy (and unspecified subsequent therapy for OS). This suggests that uptake in FDG SUVmax of greater than 5 in metastatic lobular cancer portends a poor prognosis, while FES SUVmax > 1.5 could be protective and be a predictive biomarker for endocrine based treatment response, especially when FDG uptake is not high. These results are similar to our previous study in a more heterogenous population of breast cancer lesions [36], which included patients with invasive ductal carcinoma as well as the patients with ILC analyzed here. Nevertheless, our data also suggest that the combination of FDG-PET and FES-PET will be useful in discerning disease more likely to benefit from endocrine therapy.

The cross-sectional retrospective nature of the study design is a key limitation of this analysis. The heterogeneity in disease status is both a weakness and a strength, as it suggests the utility of the imaging across both early and advanced stages of disease. Patients did not uniformly receive a contemporaneous biopsy and were at various timepoints in their metastatic cancer treatment. We acknowledge that the histology could have changed from initial biopsy site and the lack of tissue prevents evaluation of markers of endocrine resistance. Some of the studies pre-date modern therapies such as CDK4/6 inhibitors, which limits the generalizability of our observations. Early studies used older PET-reconstruction methods for validation of quantitative uptake measurements and creation of the risk classification criteria. Lesions were identified using FDG-PET and conventional imaging, so we did not assess FES-PET for detection of metastatic lesions. In addition, several patients in current, ongoing clinical trials could not be analyzed until the trials are completed. A prospective trial could address these limitations with appropriate controls, accurate assessment of histology with biopsy at time of metastases, updated PET techniques, and blinding of evaluators. The patients in our cohort were all candidates for endocrine therapy, indicating that they were not in visceral crises or critically ill. While this selects for a specific clinical group, it also supports the generalizability of our study to patients for whom FES-PET is indicated.

Integrating molecular imaging into clinical practice can improve diagnosis and treatment selection for patients. With an abundance of pharmaceutical agents available for ER + metastatic breast cancer, there is a need to identify patients most likely to benefit from different types of treatment. FES-PET has demonstrated utility as a feasible and effective predictive biomarker for clinical benefit from endocrine therapy. This clinically approved tracer is currently being tested as a predictive measure for endocrine therapy in newly diagnosed metastatic breast cancer in a multicenter phase II trial (EAI142).

In conclusion, our data shows that metastatic lobular breast cancers can be appreciated by FES and FDG PET and have similar FES and FDG avidity, with the majority of ILC metastasis showing higher FES than FDG, consistent with the strongly ER positive indolent phenotype of ILC. Bone dominant disease in patients with lobular breast cancer can be identified by FES which supports the rationale to study FES to detect bone lesions and monitor response to therapy. The combination of FES and FDG can be used to identify prognosis, select appropriate therapy and understand tumor biology. Prospective trials are needed to define the role of FES-PET in breast cancer diagnostic and treatment algorithms.

## Supplementary Information

Below is the link to the electronic supplementary material.

Supplementary file1 (DOCX 109 KB)

## Data Availability

The datasets used and/or analysed during the current study are available from the corresponding author on reasonable request.

## References

[1] Mariotto AB, Etzioni R, Hurlbert M, Penberthy L, Mayer M (2017) Estimation of the number of women living with metastatic breast cancer in the United States. Cancer Epidemiol Biomarkers Prev 26(6):809–815 28522448 10.1158/1055-9965.EPI-16-0889 PMC5833304

[2] Li CI, Anderson BO, Daling JR, Moe RE (2003) Trends in incidence rates of invasive lobular and ductal breast carcinoma. JAMA 289(11):1421–1424 12636465 10.1001/jama.289.11.1421

[3] Dossus L, Benusiglio PR (2015) Lobular breast cancer: incidence and genetic and non-genetic risk factors. Breast Cancer Res 17:37 25848941 10.1186/s13058-015-0546-7 PMC4357148

[4] García-Fernández A, Lain JM, Chabrera C, García Font M, Fraile M, Barco I, Torras M, Reñe A, González S, González C, Piqueras M (2015) Comparative long-term study of a large series of patients with invasive ductal carcinoma and invasive lobular carcinoma. Loco-regional recurrence, metastasis, and survival. Breast J 21(5):533–7 26190560 10.1111/tbj.12455

[5] Adachi Y, Ishiguro J, Kotani H, Hisada T, Ichikawa M, Gondo N, Yoshimura A, Kondo N, Hattori M, Sawaki M et al (2016) Comparison of clinical outcomes between luminal invasive ductal carcinoma and luminal invasive lobular carcinoma. BMC Cancer 16:248 27015895 10.1186/s12885-016-2275-4 PMC4807554

[6] Mouabbi JA, Hassan A, Lim B, Hortobagyi GN, Tripathy D, Layman RM (2022) Invasive lobular carcinoma: an understudied emergent subtype of breast cancer. Breast Cancer Res Treat 193(2):253–264 35347549 10.1007/s10549-022-06572-w

[7] Bos R, van Der Hoeven JJ, van Der Wall E, van Der Groep P, van Diest PJ, Comans EF, Joshi U, Semenza GL, Hoekstra OS, Lammertsma AA et al (2002) Biologic correlates of (18)fluorodeoxyglucose uptake in human breast cancer measured by positron emission tomography. J Clin Oncol 20(2):379–387 11786564 10.1200/JCO.2002.20.2.379

[8] Buck A, Schirrmeister H, Kühn T, Shen C, Kalker T, Kotzerke J, Dankerl A, Glatting G, Reske S, Mattfeldt T (2002) FDG uptake in breast cancer: correlation with biological and clinical prognostic parameters. Eur J Nucl Med Mol Imaging 29(10):1317–1323 12271413 10.1007/s00259-002-0880-8

[9] Hogan MP, Goldman DA, Dashevsky B, Riedl CC, Gönen M, Osborne JR, Jochelson M, Hudis C, Morrow M, Ulaner GA (2015) Comparison of 18F-FDG PET/CT for systemic staging of newly diagnosed invasive lobular carcinoma versus invasive ductal carcinoma. J Nucl Med 56(11):1674–1680 26294295 10.2967/jnumed.115.161455 PMC4869690

[10] National comprehensive cancer network guidelines version i.2024.invasive breast cancer. http://www.nccn.org. Accessed 11 Nov 2024

[11] Aurilio G, Disalvatore D, Pruneri G, Bagnardi V, Viale G, Curigliano G, Adamoli L, Munzone E, Sciandivasci A, De Vita F et al (2014) A meta-analysis of oestrogen receptor, progesterone receptor and human epidermal growth factor receptor 2 discordance between primary breast cancer and metastases. Eur J Cancer 50(2):277–289 24269135 10.1016/j.ejca.2013.10.004

[12] Amir E, Miller N, Geddie W, Freedman O, Kassam F, Simmons C, Oldfield M, Dranitsaris G, Tomlinson G, Laupacis A et al (2012) Prospective study evaluating the impact of tissue confirmation of metastatic disease in patients with breast cancer. J Clin Oncol 30(6):587–592 22124102 10.1200/JCO.2010.33.5232 PMC5015424

[13] Gerlinger M, Rowan AJ, Horswell S, Math M, Larkin J, Endesfelder D, Gronroos E, Martinez P, Matthews N, Stewart A et al (2012) Intratumor heterogeneity and branched evolution revealed by multiregion sequencing. N Engl J Med 366(10):883–892 22397650 10.1056/NEJMoa1113205 PMC4878653

[14] McGranahan N, Swanton C (2017) Clonal Heterogeneity and tumor evolution: past, present, and the Future. Cell 168(4):613–628 28187284 10.1016/j.cell.2017.01.018

[15] Giuliano M, Schifp R, Osborne CK, Trivedi MV (2011) Biological mechanisms and clinical implications of endocrine resistance in breast cancer. Breast 20(Suppl 3):S42-49 22015292 10.1016/S0960-9776(11)70293-4

[16] Sighoko D, Liu J, Hou N, Gustafson P, Huo D (2014) Discordance in hormone receptor status among primary, metastatic, and second primary breast cancers: biological difference or misclassification? Oncologist 19(6):592–601 24807915 10.1634/theoncologist.2013-0427 PMC4041672

[17] Ulaner GA, Vaz SC (2024) Women’s health update: growing role of pet for patients with breast cancer. Semin Nucl Med 54(2):247–255 38365547 10.1053/j.semnuclmed.2024.01.007

[18] Kurland BF, Wiggins JR, Coche A, Fontan C, Bouvet Y, Webner P, Divgi C, Linden HM (2020) Whole-body characterization of estrogen receptor status in metastatic breast cancer with 16α-18f-fluoro-17β-estradiol positron emission tomography: meta-analysis and recommendations for integration into clinical applications. Oncologist 25(10):835–844 32374053 10.1634/theoncologist.2019-0967 PMC7543360

[19] Peterson LM, Kurland BF, Yan F, Jiresova AN, Gadi VK, Specht JM, Gralow JR, Schubert EK, Link JM, Krohn KA et al (2021) F-fluoroestradiol PET imaging in a Phase II trial of vorinostat to restore endocrine sensitivity in ER+/HER2- metastatic breast cancer. J Nucl Med 62(2):184–190 32591490 10.2967/jnumed.120.244459 PMC9364869

[20] Lindström LS, Yau C, Czene K, Thompson CK, Hoadley KA, Van’t Veer LJ, Balassanian R, Bishop JW, Carpenter PM, Chen YY et al (2018) Intratumor heterogeneity of the estrogen receptor and the long-term risk of fatal breast cancer. J Natl Cancer Inst 110(7):726–733 29361175 10.1093/jnci/djx270 PMC6037086

[21] Almendro V, Kim HJ, Cheng YK, Gönen M, Itzkovitz S, Argani P, van Oudenaarden A, Sukumar S, Michor F, Polyak K (2014) Genetic and phenotypic diversity in breast tumor metastases. Cancer Res 74(5):1338–1348 24448237 10.1158/0008-5472.CAN-13-2357-T PMC3963810

[22] Mortimer JE, Dehdashti F, Siegel BA, Trinkaus K, Katzenellenbogen JA, Welch MJ (2001) Metabolic flare: indicator of hormone responsiveness in advanced breast cancer. J Clin Oncol 19(11):2797–2803 11387350 10.1200/JCO.2001.19.11.2797

[23] Linden HM, Stekhova SA, Link JM, Gralow JR, Livingston RB, Ellis GK, Petra PH, Peterson LM, Schubert EK, Dunnwald LK et al (2006) Quantitative fluoroestradiol positron emission tomography imaging predicts response to endocrine treatment in breast cancer. J Clin Oncol 24(18):2793–2799 16682724 10.1200/JCO.2005.04.3810

[24] Dehdashti F, Mortimer JE, Trinkaus K, Naughton MJ, Ellis M, Katzenellenbogen JA, Welch MJ, Siegel BA (2009) PET-based estradiol challenge as a predictive biomarker of response to endocrine therapy in women with estrogen-receptor-positive breast cancer. Breast Cancer Res Treat 113(3):509–517 18327670 10.1007/s10549-008-9953-0 PMC3883567

[25] Peterson LM, Kurland BF, Schubert EK, Link JM, Gadi VK, Specht JM, Eary JF, Porter P, Shankar LK, Mankoff DA et al (2014) A phase 2 study of 16α-[18F]-fluoro-17β-estradiol positron emission tomography (FES-PET) as a marker of hormone sensitivity in metastatic breast cancer (MBC). Mol Imaging Biol 16(3):431–440 24170452 10.1007/s11307-013-0699-7 PMC4169237

[26] Boers J, Venema CM, de Vries EFJ, Glaudemans AWJM, Kwee TC, Schuuring E, Martens JWM, Elias SG, Hospers GAP, Schröder CP (2020) Molecular imaging to identify patients with metastatic breast cancer who benefit from endocrine treatment combined with cyclin-dependent kinase inhibition. Eur J Cancer 126:11–20 31891878 10.1016/j.ejca.2019.10.024

[27] Venema C, de Vries E, Glaudemans A, Poppema B, Hospers G, Schröder C (2017) 18F-FES PET has added value in staging and therapy decision making in patients with disseminated lobular breast cancer. Clin Nucl Med 42(8):612–614 28604479 10.1097/RLU.0000000000001724

[28] Ulaner GA, Jhaveri K, Chandarlapaty S, Hatzoglou V, Riedl CC, Lewis JS, Mauguen A (2021) Head-to-head evaluation of 18F-FES and 18F-FDG PET/CT in metastatic invasive lobular breast cancer. J Nucl Med 62(3):326–331 32680923 10.2967/jnumed.120.247882 PMC8049349

[29] Liu C, Ma G, Xu X, Song S, Yang Z (2024) Can 18F-FES PET Improve the Evaluation of 18F-FDG PET in patients with metastatic invasive lobular carcinoma? Clin Nucl Med 49(4):301–307 38427956 10.1097/RLU.0000000000005085

[30] Covington MF, Hoffman JM, Morton KA, Buckway B, Boucher KM, Rosenthal RE, Porretta JM, Brownson KE, Matsen CB, Vaklavas C et al (2023) Prospective Pilot Study of (18)F-Fluoroestradiol PET/CT in patients with invasive lobular carcinomas. AJR Am J Roentgenol 221(2):228–239 36919879 10.2214/AJR.22.28809

[31] Byrd DW, Doot RK, Allberg KC, MacDonald LR, McDougald WA, Elston BF, Linden HM, Kinahan PE (2016) Evaluation of cross-calibrated (68)Ge/(68)Ga phantoms for assessing PET/CT measurement bias in oncology imaging for single- and multicenter trials. Tomography 2(4):353–360 28066807 10.18383/j.tom.2016.00205 PMC5214172

[32] Kurland BF, Peterson LM, Shields AT, Lee JH, Byrd DW, Novakova-Jiresova A, Muzi M, Specht JM, Mankoff DA, Linden HM et al (2019) Test-retest reproducibility of (18)F-FDG PET/CT uptake in cancer patients within a qualified and calibrated local network. J Nucl Med 60(5):608–614 30361381 10.2967/jnumed.118.209544 PMC6495239

[33] Tewson TJ, Mankoff DA, Peterson LM, Woo I, Petra P (1999) Interactions of 16alpha-[18F]-fluoroestradiol (FES) with sex steroid binding protein (SBP). Nucl Med Biol 26(8):905–913 10708304 10.1016/s0969-8051(99)00072-4

[34] Ulaner GA, Mankoff DA, Clark AS, Fowler AM, Linden HM, Peterson LM, Dehdashti F, Kurland BF, Mortimer J, Mouabbi J et al (2023) Summary: appropriate use criteria for estrogen receptor-targeted PET imaging with 16alpha-(18)f-fluoro-17beta-fluoroestradiol. J Nucl Med 64(3):351–354 36863779 10.2967/jnumed.123.265420

[35] Peterson LM, Kurland BF, Link JM, Schubert EK, Stekhova S, Linden HM, Mankoff DA (2011) Factors influencing the uptake of 18F-fluoroestradiol in patients with estrogen receptor positive breast cancer. Nucl Med Biol 38(7):969–978 21982568 10.1016/j.nucmedbio.2011.03.002 PMC4108284

[36] Kurland BF, Peterson LM, Lee JH, Schubert EK, Currin ER, Link JM, Krohn KA, Mankoff DA, Linden HM (2017) Estrogen receptor binding (18F-FES PET) and Glycolytic Activity (18F-FDG PET) predict progression-free survival on endocrine therapy in patients with ER+ breast cancer. Clin Cancer Res 23(2):407–415 27342400 10.1158/1078-0432.CCR-16-0362 PMC5183531

[37] de Mooij CM, Mitea C, Mottaghy FM, Smidt ML, van Nijnatten TJA (2021) Value of (18)F-FDG PET/CT for predicting axillary pathologic complete response following neoadjuvant systemic therapy in breast cancer patients: emphasis on breast cancer subtype. EJNMMI Res 11(1):116 34807395 10.1186/s13550-021-00861-z PMC8609064

[38] Han S, Lee SB, Gong G, Lee J, Chae SY, Oh JS, Moon DH (2023) Prognostic significance of pretreatment 18F-fluorodeoxyglucose positron emission tomography/computed tomography in patients with T2N1 hormone receptor-positive, ERBB2-negative breast cancer who underwent adjuvant chemotherapy. Breast Cancer Res Treat 198(2):207–15 36633721 10.1007/s10549-022-06852-5

[39] Kurland BF, Oesterreich S (2018) Heterogeneity in metastatic breast cancer. J Nucl Med 59(8):1210–1211 29903931 10.2967/jnumed.118.214304 PMC6071507

[40] Pestalozzi BC, Zahrieh D, Mallon E, Gusterson BA, Price KN, Gelber RD, Holmberg SB, Lindtner J, Snyder R, Thürlimann B et al (2008) Distinct clinical and prognostic features of infiltrating lobular carcinoma of the breast: combined results of 15 international breast cancer study group clinical trials. J Clin Oncol 26(18):3006–3014 18458044 10.1200/JCO.2007.14.9336

[41] Di Meglio A, Freedman RA, Lin NU, Barry WT, Metzger-Filho O, Keating NL, King TA, Sertoli MR, Boccardo F, Winer EP et al (2016) Time trends in incidence rates and survival of newly diagnosed stage IV breast cancer by tumor histology: a population-based analysis. Breast Cancer Res Treat 157(3):587–596 27271765 10.1007/s10549-016-3845-5

[42] Zhang J, Jia Z, Ragaz J, Zhang YJ, Zhou M, Zhang YP, Li G, Wang BY, Wang ZH, Hu XC (2013) The maximum standardized uptake value of 18 F-FDG PET scan to determine prognosis of hormone-receptor positive metastatic breast cancer. BMC Cancer 13:42 23368410 10.1186/1471-2407-13-42 PMC3583732

[43] Taghipour M, Wray R, Sheikhbahaei S, Wright JL, Subramaniam RM (2016) FDG avidity and tumor burden: survival outcomes for patients with recurrent breast cancer. AJR Am J Roentgenol 206(4):846–855 27003053 10.2214/AJR.15.15106

[44] Cachin F, Prince HM, Hogg A, Ware RE, Hicks RJ (2006) Powerful prognostic stratification by [18F]fluorodeoxyglucose positron emission tomography in patients with metastatic breast cancer treated with high-dose chemotherapy. J Clin Oncol 24(19):3026–3031 16717291 10.1200/JCO.2005.04.6326

