# Supplementary material for: [18 F]-Fluoroestradiol PET (FES-PET) and [18 F] Flurodeoxyglucose PET (FDG-PET) Imaging May Aid in Managing Therapy in Patients with Metastatic Lobular Breast Cancer
Source: Mol Imaging Biol. 2025 May 14;27(3):410–20. doi: 10.1007/s11307-025-02015-2 (PMC12162187; doi:10.1007/s11307-025-02015-2)
Supplement: Supplementary file 1 — Supplementary file1 (DOCX 109 KB) [file 11307_2025_2015_MOESM1_ESM.docx]

**[^18^F]-Fluoroestradiol PET (FES-PET) and [^18^F]-Fluorodeoxyglucose PET (FDG-PET) imaging may aid in managing therapy in patients with metastatic lobular breast cancer**

Poorni M Manohar^1,2^, Lanell M Peterson^1,2^, Isaac C Jenkins^2^, Qian (Vicky) Wu^2^, Brenda F Kurland^3^, Alena Novakova-Jiresova^4^, Mark Muzi^1^, Delphine L Chen^1,2^, Jennifer M Specht^1,2^, Suzanne Dintzis^1,2^, Paul E Kinahan^1,2^, David A Mankoff^5^, and Hannah M Linden^1,2​^

**Supplemental Fig 1** FES and FDG SUVmax values per lesion showing lesion heterogeneity within each patient. Each panel is an individual patient. Diamonds indicate average SUVmax and the patient’s assignment.

**Supplemental Table 1** Comparison of FES and FDG uptake in patients with bone dominant and bone marrow uptake.

|  | FES uptake mean (95% Cl) | FDG uptake mean (95%Cl) | FES p value (bone dominant vs bone marrow) | FDG p value (bone dominant vs bone marrow) |
| --- | --- | --- | --- | --- |
| Bone dominant (n=15) | 3.17 (2.47, 4.06) | 4.34 (3.43, 5.49) | 0.16 | .054 |
| Bone marrow (n=3) | 4.66 (2.72, 7.99) | 5.11 (3.06, 8.53) |  |  |
